# REG3β modifies cell tumor function by impairing extracellular vesicle uptake

**DOI:** 10.1038/s41598-017-03244-4

**Published:** 2017-06-09

**Authors:** Laia Bonjoch, Meritxell Gironella, Juan Lucio Iovanna, Daniel Closa

**Affiliations:** 1 Dept Experimental Pathology, Institut d’Investigacions Biomèdiques de Barcelona-Consejo Superior de Investigaciones científicas (IIBB-CSIC), Institut d’Investigacions Biomèdiques August Pi i Sunyer (IDIBAPS), Barcelona, 08036 Spain; 2 Gastrointestinal and Pancreatic Oncology, Hospital Clínic de Barcelona, Centro de Investigación Biomédica en Red de Enfermedades Hepáticas y Digestivas (CIBEREHD), Institut d’Investigacions Biomèdiques August Pi i Sunyer (IDIBAPS), Barcelona, 08036 Spain; 3 Centre de Recherche en Cancérologie de Marseille (CRCM), Institut National De La Santé Et De La Recherche Médicale (INSERM) Unit 1068, Centre National De La Recherche Scientifique (CNRS) Unit 7258, Aix-Marseille Université and Institut Paoli-Calmettes, 13273 Marseille, Cedex 09 France

## Abstract

Extracellular vesicles (EVs), including exosomes and microvesicles, are nano-sized membrane vesicles containing proteins and nucleic acids, which act as intercellular messengers. They play an important role in a variety of physiological processes, as well as in pathological situations such as inflammation or cancer. Here, we show that in the case of pancreatic ductal adenocarcinoma (PDAC), the healthy pancreatic tissue surrounding the tumor releases REG3β, a lectin that binds to the glycoproteins present in the surface of EVs, thus interfering with their uptake and internalization by target cells. *In vitro*, the disruption of the signaling mediated by EVs due to the presence of REG3β, prevents the EV-induced phenotypic switch in macrophages, inhibits the increased cell migration of cancer cells and reverses a number of metabolomic changes promoted by EVs. *In vivo*, the uptake of REG3β^+^ EVs by tumor cells is significantly impaired. Furthermore, it results in an increase of circulating REG3β^+^ EVs in blood of pancreatic cancer patients. Our findings highlight the effect of a lectin released by the healthy pancreatic tissue surrounding the tumor in modulating the EV-mediated interactions between different cell types in PDAC.

## Introduction

Extracellular vesicles (EVs), including exosomes and microvesicles, are nano-sized membrane vesicles containing proteins and nucleic acids that act as intercellular messengers^1^. Initially considered cellular waste product membranes, it is now clear that they play an important role as mediators of intercellular communication in many physiological and pathological processes, particularly in inflammation and cancer^2^. In the case of pancreatic ductal adenocarcinoma (PDAC), one of the most lethal cancers, there is an increase in the concentration of circulating EVs in plasma^3^ that has been linked to the formation of liver metastases^4^. The origin of these EVs has not been well-defined, but it is thought to be caused by an increased synthesis by cancer cells and other cells of the tumor stroma.

It is clear that EVs are very actively involved in intercellular communication within the tumor, but their specific effects are still little understood. For example, it has been described that they can regulate the immune response, either by inducing tolerance to tumors^5, 6^ or by generating anti-tumor immune responses^7^. Although these opposing results can be explained in part by differences in experimental designs, the potential effect of soluble mediators in interfering with or promoting EV-mediated signaling must be considered. The EV uptake by target cells involves different mechanisms, some of them dependent on glycoproteins present in the membrane^8–10^. Consequently, interactions between these glycoproteins and soluble lectins released into the intercellular compartment can play a prominent role in the fate of EVs. This could be particularly important in PDAC since acinar cells are a well-known source of REG3β, a soluble C-type lectin also known as PAP or HIP, which is released in high amounts in the course of pancreatic inflammation, cell injury and pancreatic cancer^11^.

Here we show that REG3β binds the surface of EVs through its lectin domain, thereby interfering with the internalization of EVs by target cells and compromising their functionality. This mechanism could be important in PDAC, since we detected REG3β^+^ EVs in blood from PDAC patients.

## Results

### REG3β inhibits the uptake of EVs both *in vitro* and *in vivo*

**Figure 1:**
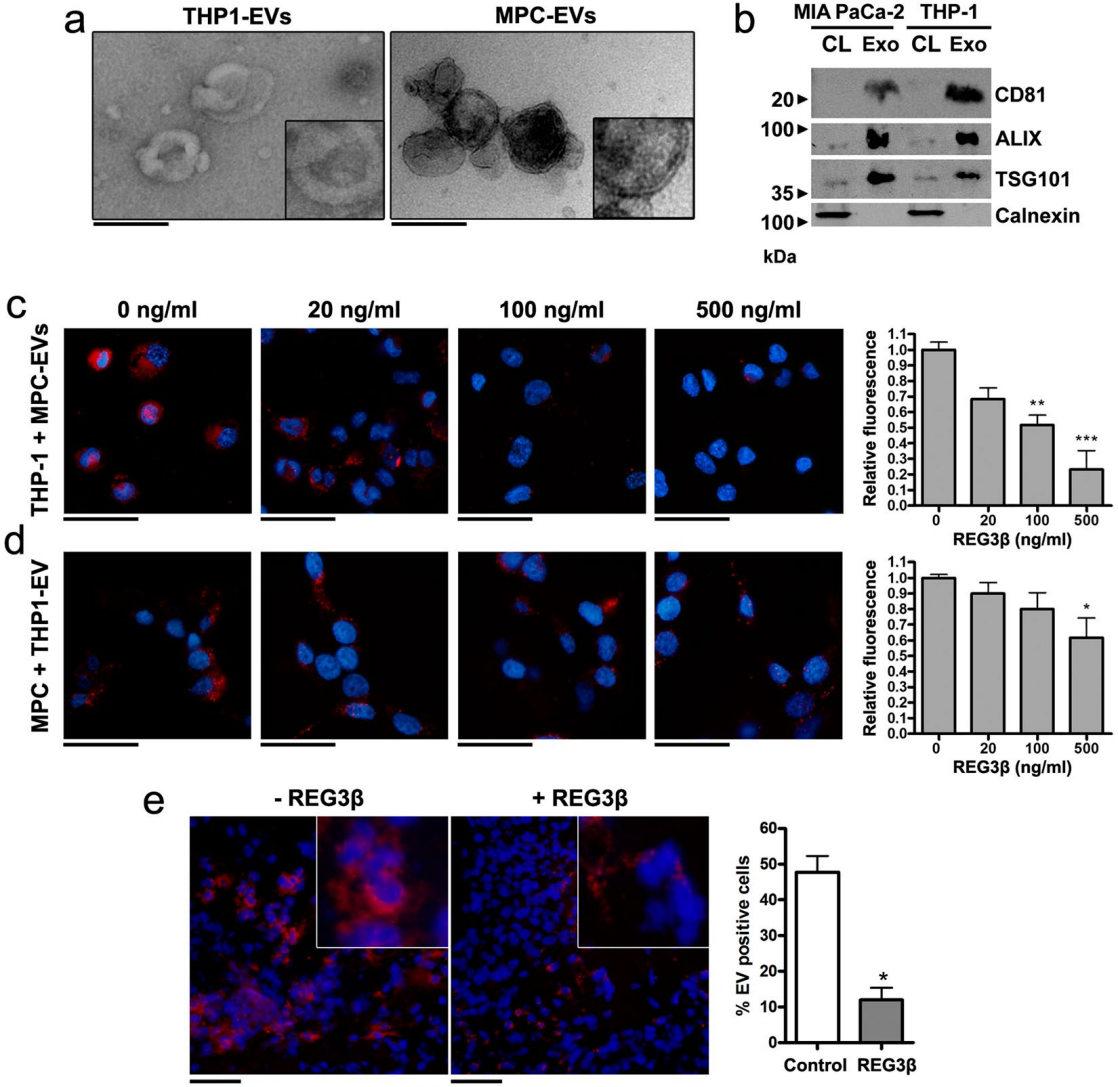
REG3β inhibits the uptake of EVs both *in vitro* and *in vivo*. (**a**) Transmission electron microscopy images of 120,000 × g pelleted THP1-EVs and MPC-EVs. 2x magnification in the lower right corner to appreciate the double membrane. Scale bars: 200 nm. (**b**) Representative Western blot of EVs samples and cell lysates to confirm the presence of classical exosome markers (CD81, ALIX, TSG101) and the absence of endoplasmic reticulum contamination (Calnexin). (**c,d**) Fluorescence microscopy of THP-1 macrophages (**c**) and MIA PaCa-2 cells (MPC) (**d**) incubated, respectively, with 3 µg/ml of PKH26-labeled MPC EVs or THP1-EVs and increasing concentrations of REG3β. Nuclei counterstained with DAPI. On the right, quantification of the amount of EVs internalization via fluorimetric reading (n = 4). Data are expressed as mean ± SEM. *P < 0.05, **P < 0.01, ***P < 0.001, compared to 0 ng/ml REG3β. ANOVA with Tukey’s post-test was used to calculate *P*-values. Scale bars: 50 µm. (**e**) PKH26-labeled EVs injected into tumor xenografts were uptaken by tumor cells (−REG3β) but remained in the intercellular space when pretreated with REG3β (+REG3β). Nuclei counterstained with DAPI. 4x magnification in the top right corner. Scale bars: 50 µm.

Transmission electron microscopy characterization of EVs from THP-1 macrophages and MIA PaCa-2 cells (MPC) revealed a size ranging from 50 to 150 nm of diameter and the presence of a lipid bilayer (Fig. 1a). The concomitant expression of CD81 membrane protein with the endosomal markers TSG101 and ALIX (Fig. 1b) confirmed the correct purification of EVs and that they were enriched in exosomes^12^. These markers were almost not detectable on the same amount of protein from cell lysates, reinforcing the lack of contamination from cell debris. In addition, the absence of Calnexin, an endoplasmic reticulum marker, confirmed that the EV population was not contaminated with vesicles from other cellular compartments.

The initial *in vitro* experiments were designed to test the effect of REG3β on the EV-mediated signaling between inflammatory and tumor cells. We first measured, in macrophage-differentiated THP-1 cells, the uptake of PKH26-stained EVs purified from the pancreatic cancer cell line MIA PaCa-2 (MPC-EVs). MPC-EV internalization on macrophages was clearly visible and was inhibited by REG3β in a dose dependent manner (Fig. 1c). This interfering effect does not seem to be a cell-specific mechanism, since a similar result was observed in the opposite experiment, evaluating MIA PaCa-2 uptake of EVs purified from THP-1 cells (THP1-EVs) (Fig. 1d). Although the phagocytic capability of these cells is lower than that of macrophages, similar dose-dependent inhibition of EV capture was also observed in the presence of REG3β.

We then examined whether cellular uptake of EVs in the tumor microenvironment *in vivo* could be also impaired by the presence of REG3β. For this purpose, MIA PaCa-2 cells were transplanted subcutaneously into BALB/C *nude* mice. We took advantage of the fact that MIA PaCa-2 cells do not express REG3β and that the subcutaneous localization of xenografts prevented the presence of REG3β generated by the healthy acinar pancreatic cells. In this case, EVs were previously treated with REG3β, ensuring that the observed effects were exclusively due to the interactions of this protein with EVs instead of with target cells. Four weeks after cell injection, when tumor xenografts were established, REG3β-treated and non-treated EVs purified from cultured MIA PaCa-2 cells were stained and injected into the tumors. One hour later, we obtained the tumors and analyzed the location of EVs by fluorescent microscopy. As observed *in vitro*, tumor cells in tumor xenografts showed a high uptake of fluorescent-stained EVs, while the REG3β treatment significantly impaired the incorporation of EVs into recipient cells and remained in the intercellular space (Fig. 1e).

### REG3β interacts with EVs through its lectin domain

**Figure 2:**
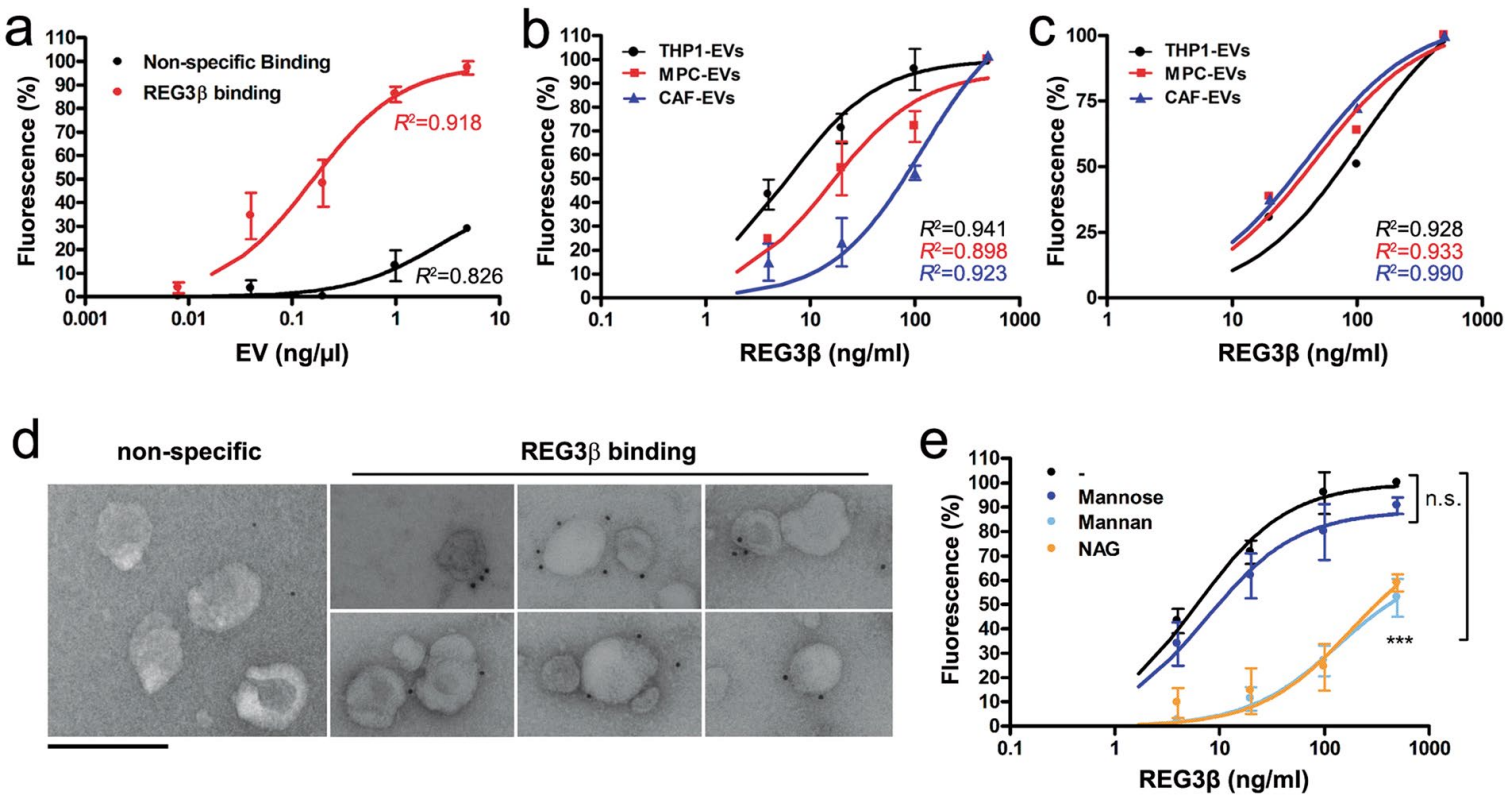
REG3β interacts with EVs through its lectin domain. (**a**) PKH26-labeled THP1-EVs binding to plates coated with anti-REG3β antibody and saturated with 500 ng/ml of REG3β. The indicated concentrations of EVs were used. Non-specific binding was assessed in the absence of REG3β. Data are depicted as relative expression to saturation (5 ng/µl of EV) (n = 4). (**b**,**c**) PKH26-labeled EVs (1 ng/µl of EV protein) binding to plates (**b**) or magnetic beads (**c**) coated with anti-REG3β antibody and incubated with the indicated concentrations of REG3β. Data are depicted as relative expression to maximum binding (500 ng/ml of REG3β). (**d**) Anti-REG3β immunogold labeling of THP1-EVs incubated with REG3β (500 ng/ml). Non-specific binding was tested in the absence of REG3β. Scale bar: 200 nm. (**e**) Competitive assay of PKH26-labeled THP1-EVs binding to REG3β as in (B), but in the presence of 1 mg/ml mannose, 1 mg/ml mannan or 5 mM of NAG. (n = 4). ***P < 0.001 compared to non-sugar binding (F test). In all panels, data are expressed as mean ± SEM. *R* ^2^ represents goodness of fit to the one-site binding hyperbola model.

On the basis of these results, we hypothesized that REG3β interferes with the uptake of EVs through the binding to their membrane glycoproteins. To verify this hypothesis we designed an immunoassay coating a multiwell plate with rabbit anti-human REG3β antibody. In the first experiment, we saturated the plate with REG3β, and increasing amounts of PKH26-labeled THP1-EVs were added. After washing, fluorescence intensity levels revealed a dose dependent binding that achieved a maximum when the concentration of EVs corresponded to 1 ng/µl of EV protein (Fig. 2a). Thus, this EV concentration was selected in the subsequent assays. In the second experiment, increasing concentrations of REG3β (from 0 to 500 ng/ml) and a fixed amount of labeled EVs (1 ng protein/µl) were added to the plate. After washing the unbound EVs, the fluorescence measurement showed a clear dose response that was dependent on the amount of REG3β, indicating that the EV retention was effectively mediated by their interaction with REG3β (Fig. 2b). This binding was observed not only in THP1-EVs and MPC-EVs, but also in EVs purified from cancer-associated fibroblasts (CAF-EVs), suggesting the non-specific nature of the REG3β-EV interaction. An additional experiment was carried out to further confirm this interaction. In this case, we used magnetic beads coated with anti-REG3β antibody to purify labeled EVs obtained from the same three cell populations (Fig. 2c). Once again, the recovery of EVs was dependent on the amount of REG3β added to the system.

To further demonstrate the interaction between EVs and REG3β, THP1-EVs treated with REG3β were analyzed via immunoelectron microscopy using a specific anti-REG3β antibody and a 12 nm gold-conjugated secondary antibody (Fig. 2d). In the absence of REG3β, very little labeling was observed, similar to the samples from which the primary antibody had been omitted. By contrast, specific immunogold labeling was apparent on the surface of EVs treated with REG3β.

Finally, a sugar-based competitive assay was developed in order to verify the lectin nature of the interaction between EVs and REG3β. It has been reported that although REG3β binds to complex sugars, such as N-Acetyl-D-glucosamine (NAG) or mannan (a polysaccharide composed of polymerized mannose), it does not bind to monomeric mannose^13^. Along this line, we found that mannose did not modify the binding capacity of THP1-EVs to REG3β. However, in the presence of mannan and NAG, the interaction between EVs and REG3β was significantly blocked, so that even when plates were saturated with REG3β only 60% of maximum binding was reached (Fig. 2e).

### REG3β-blocked EVs lose their capability to modulate some cell functions

Once demonstrated that REG3β prevented the capture of EVs by target cells, we aimed to investigate whether impaired signal transduction through EV blockade could have functional consequences affecting the different cell populations present in tumor microenvironment.

**Figure 3:**
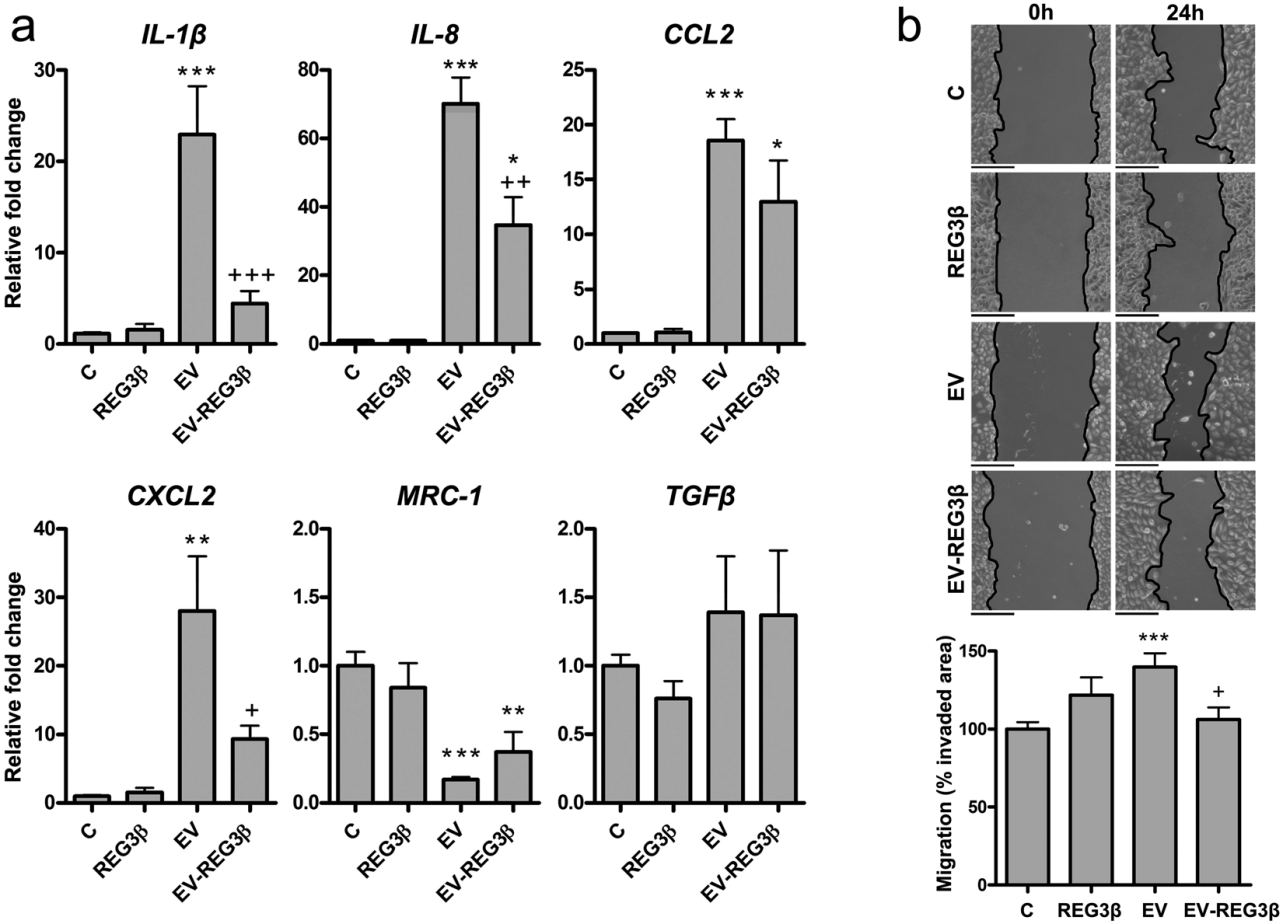
EVs blocked by REG3β lose their capability to modulate some cell functions. (**a**) qPCR analysis of different pro-inflammatory *(IL-1β, IL-8, CCL2, CXCL2*) and anti-inflammatory/regulatory (*MRC-1, TGFβ*) markers on THP-1 macrophages treated with 500 ng/ml of REG3β, and 3 µg/ml of MPC-EVs (EV) or REG3β-blocked MPC-EVs (EV-REG3β) for 24 h (n = 6). Data are depicted as relative expression to the *GAPDH* housekeeping gene. (**b**) Scratch-wound healing assay of MIA PaCa-2 cells incubated with 500 ng/ml of REG3β, and 3 µg/ml of THP1-EVs (EV) or REG3β-blocked EVs (EV-REG3β) for 24 h (n = 6). Down, quantification of cell migration. Scale bars: 200 µm. In all panels, data are expressed as mean ± SEM. ANOVA with Tukey’s post-test was used to calculate *P*-values. *P < 0.05, **P < 0.01, ***P < 0.001 compared to non-treated (C), ^+^ *P* < 0.05, ^++^ *P* < 0.01, ^+++^ *P* < 0.001 compared to EV.

We first analyzed the capability of REG3β-blocked MPC-EVs to modulate the phenotypic switch of macrophages. EVs generated by cancer cells promoted the polarization of macrophages to an inflammatory phenotype, characterized by the upregulation of several M1-associated genes (*IL-1β, IL-8, CCL2, CXCL2*) and the downregulation of *MRC1* expression, an M2-related marker. However, the presence of REG3β-blocked EVs prevented the polarization role of EV on macrophages (Fig. 3a). Similar effects were observed when we evaluated tumor cell migration through a scratch-wound healing assay. THP1-EVs increased MIA PaCa-2 cell migration, but when those EVs were pretreated with REG3β, they lost their capacity to stimulate tumor cell migration (Fig. 3b).

**Figure 4:**
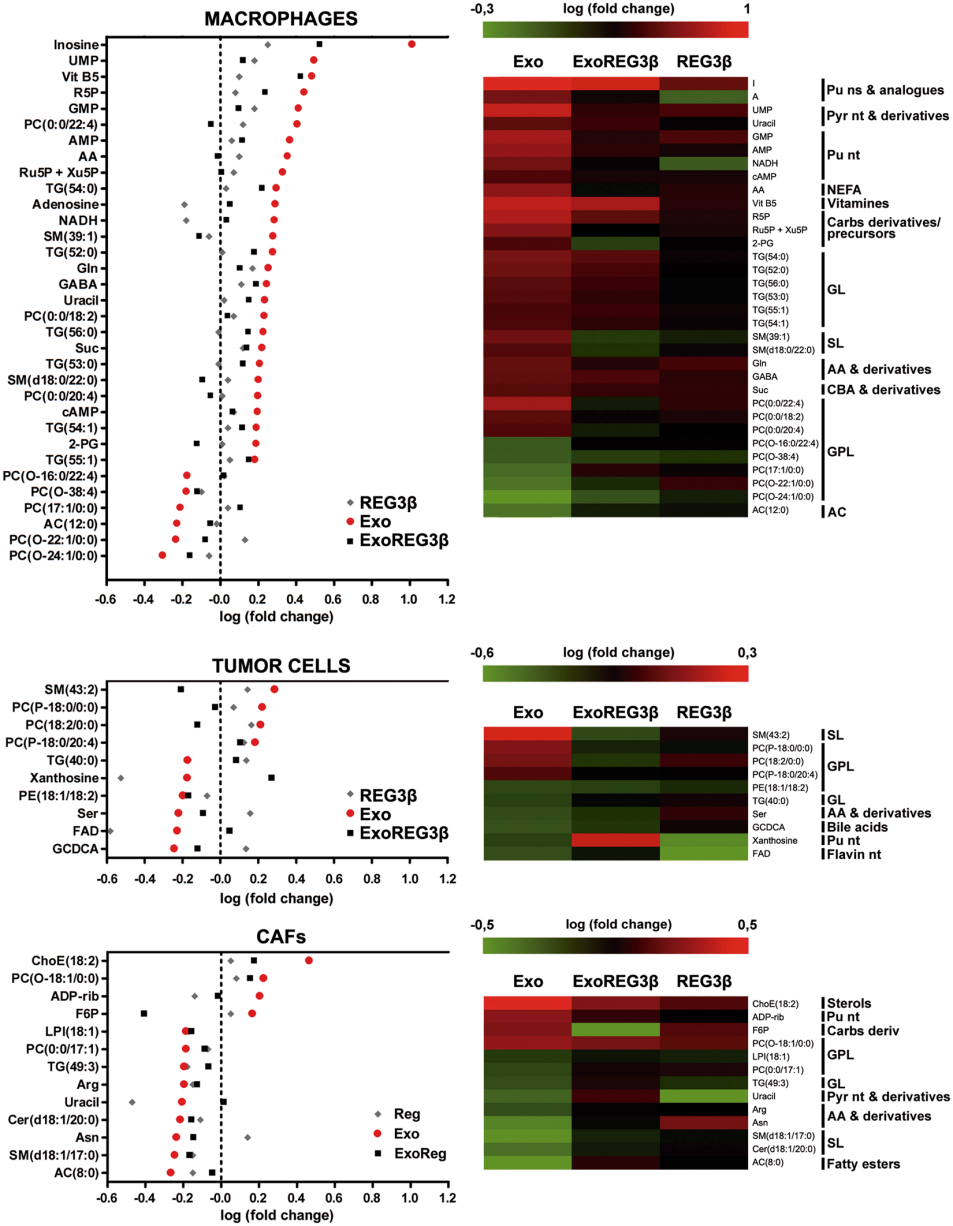
Impaired metabolic switch in the three main cell types of tumor microenvironment due to EV blockage by REG3β. Metabolomic analysis of those metabolites which the REG3β-blocking of signal transduction through EVs prevented increases or decreases in their dysregulation. Heatmaps of metabolites grouped according to their chemical class in order to illustrate the most frequent altered families by this blocking mechanism. AA (aminoacids), AC (acylcarnitines), Carbs (carbohydrates), CBA (carboxylic acids), GL (glycerolipids), GPL (glycerophospholipids), NEFA (non-esterified fatty acids), ns (nucleosides), nt (nucleotides), Pu (purines), Pyr (pyrimidines), SL (sphingolipids).

Finally, we performed a metabolomics assay in order to identify those changes that were caused by EVs and reverted by REG3β binding. Cancer cells have altered metabolic processes which have been reported to promote tumor growth and metastasis. Although the interaction between tumor stroma and cancer cells has not been completely elucidated, a recent work has unmasked a possible role of exosomes in cancer metabolism modulation^14^. Experiments were performed on macrophages and CAFs both treated with MPC-EVs, as well as on tumor cells treated with THP1-EV. Predictably, each cell type showed a different metabolomic profile in response to EVs, but in most cases the metabolic switch was interfered by the REG3β blocking effect on EVs. Regarding macrophages, EVs blocked with REG3β prevented increases in the concentration of different metabolites, whereas in tumor cells and CAFs changes were less notable and mostly related with preventing decreases of metabolites. Grouping metabolites according to their chemical class reflected that most of which were dysregulated after EVs treatment had been previously reported to be altered in cancer^15^ and macrophage polarization^16^, especially nucleic acids, amino acids and lipid mediators (Fig. 4).

Therefore, these results suggest that regardless of the role played by EVs in different cell systems, the presence of REG3β is associated with a loss of EVs function, probably related to the impaired cell uptake.

### REG3β-mediated EV blockage triggers REG3β^+^ EVs release to bloodstream in PDAC patients

**Figure 5:**
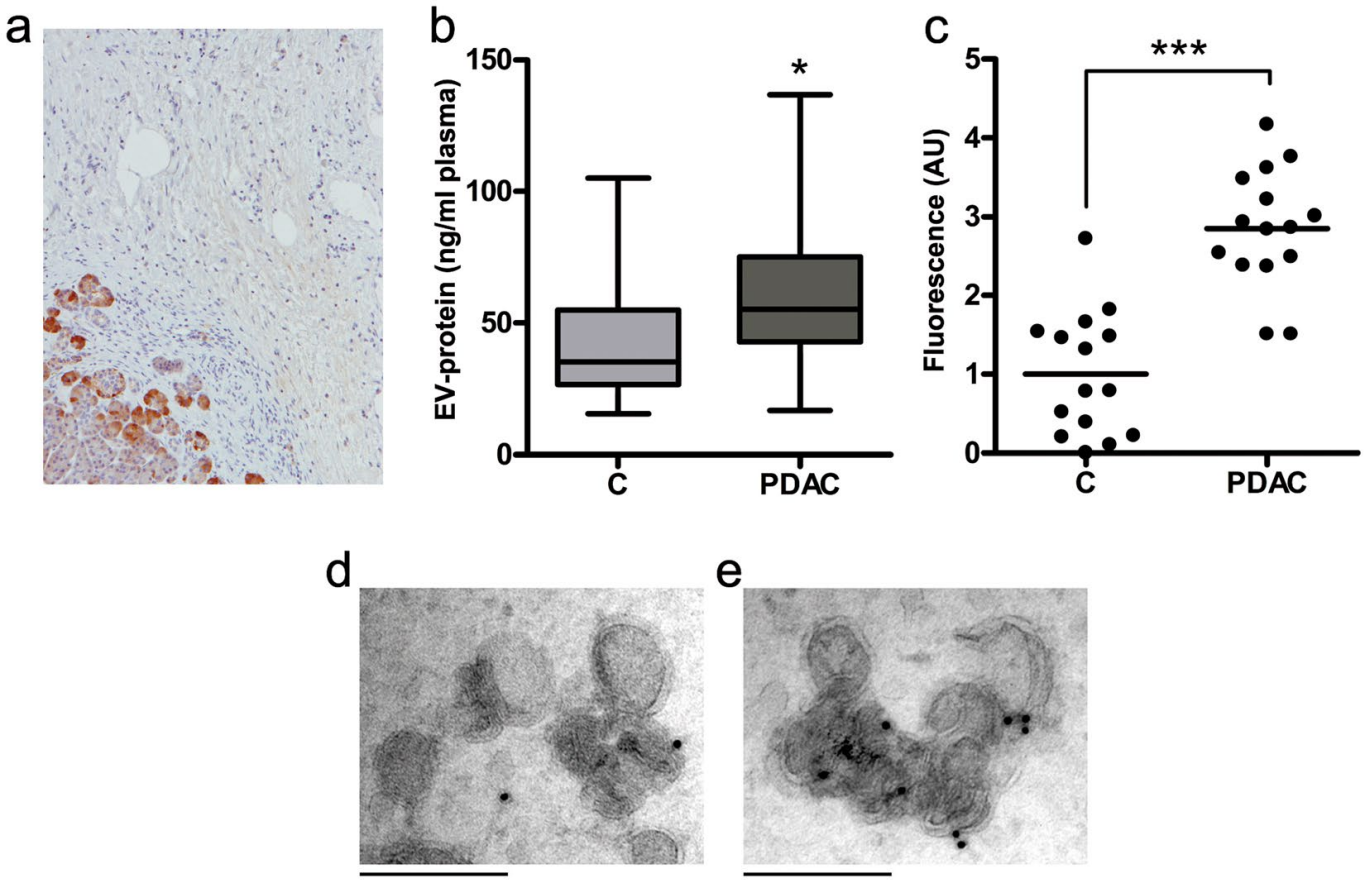
REG3β^+^ EVs are released to bloodstream in PDAC patients. (**a**) Immunolocalization by immunohistochemistry of REG3β in human PDAC. REG3β is absent in tumor cells but strongly expressed by the healthy acinar cells surrounding the tumor. (**b**) Quantification of EV levels in plasma through a Bradford assay. Circulating EVs were isolated from samples of healthy donors (C) (n = 15) and PDAC patients (n = 15). Data are expressed as mean ± SEM. Student’s t-test used to calculate *P*-values. *P < 0.05. (**c**) Human PKH26-labeled EVs (1 ng/µl of EV protein) binding to plates coated with anti-REG3β antibody. Data are depicted as PDAC EVs binding levels relative to healthy donors samples. Significance determined using a two-tailed Mann–Whitney U test. ***P < 0.001. (**d**,**e**) Representative immunogold labeling of EVs from healthy donors (D) or PDAC patients (E) with anti-REG3β. Scale bars: 200 nm.

The histochemical analysis of PDAC samples from human patients revealed high protein levels of REG3β restricted to the healthy pancreatic acinar cells surrounding the tumor, being this distant microenvironment the major source of REG3β in pancreatic cancer (Fig. 5a). The release of REG3β around the tumor suggests that, as we observed in xenografts, uptake of EVs generated inside the tumor could be impaired due to the blocking effect of this secreted lectin. Consequently, a relevant release of REG3β^+^ EVs to the bloodstream could be expected in patients with pancreatic cancer. We checked the amount of circulating EVs in a cohort of healthy donors and pancreatic cancer patients and we effectively detected a significant increase in the number of EVs present in the plasma of PDAC patients (Fig. 5b). Moreover, when the same amounts of EVs were added to anti-REG3β coated plates, we confirmed that the majority of EVs associated with pancreatic cancer samples were REG3β^+^ (Fig. 5c). This observation was further supported by immunogold labeling and electron microscopy image acquisition of EVs from healthy donors (Fig. 5d) or pancreatic cancer patients (Fig. 5e).

## Discussion

In this study we demonstrate that the presence of REG3β modifies the physiological role of EVs by interfering on their uptake by target cells. In the particular case of PDAC, the release of REG3β by healthy cells surrounding the tumor points to an important role for this lectin as a regulator of EV signaling.

Previous studies had already described the high degree of glycosylation of EV membranes^8, 9^ and the ability of lectins to bind to their saccharide residues^17, 18^, so the interaction of EVs with soluble lectins could be suspected. Our initial results are consistent with this hypothesis, since the presence of REG3β blocked the internalization of EVs through a non-specific mechanism, interfering in both macrophage and epithelial cell uptake. In addition, it has been previously reported that REG3β shows ligand preference for NAG and polymers of mannose as occurs with mannose-binding lectin (MBL), a C-type lectin with an established role in innate immunity^19^, and our results indicate that the same sugar residues interfere with the binding between REG3β and EV.

The membrane of EVs has a complex pattern of glycosylation^8, 9^ among which glycoproteins and glycolipids enriched in NAG and polymers of mannose have been identified^20^. These sugar residues can interact with C-type lectin cell receptors such as mannose-binding lectin (MBL), a C-type lectin with an established role in innate immunity, and also with DEC-205 and DC-SIGN, which have been reported to be involved in EV uptake^21^. Some works have depicted that blocking these receptors drastically reduces EV uptake^10^, highlighting the importance of these sugar residues on EV capture.

On the other hand, REG3β is a small protein composed only by a single lectin domain and a short N-terminal peptide, which drives its secretion^22^. It has been previously reported that REG3β shows ligand preference for NAG and polymers of mannose^19^. Taking into account REG3β structure and the ability of other soluble lectins to bind to EV saccharide residues^17, 18^, the interaction of EVs with REG3β could be suspected. Our results showed that the presence of REG3β blocked the internalization of EVs through a non-specific mechanism, interfering in both macrophage and epithelial cell uptake. We demonstrated the interaction between REG3β and EVs and confirmed that NAG and mannan interfere on this interaction. For this reason, we conclude that sugars to which REG3β binds are relevant for EV capture and that the lectin nature of REG3β drives this process. However, further studies are needed to better understand the specific interaction between REG3β and the EV glycome. In addition, as the glycosylation pattern of EVs can change according to the context in which they are secreted, the glycome analysis would allow elucidating the importance of REG3β in different circumstances.

The role of EV signaling in cancer cell migration and metastasis is unclear and a number of opposite results have been reported. Nevertheless, the presence of REG3β modifying the interaction of EVs with target cells could be an additional explanation to understand these conflicting results. REG3β has been reported to be an anti-inflammatory acute phase protein generated by the pancreas and other organs in response to stress. Taking into account the potential danger of hydrolytic enzymes stored in pancreatic acinar cells, the generation of large amounts of REG3β seems to be an appropriate response during pancreatic inflammation. In this line, it is not surprising that REG3β, normally absent in control conditions, becomes the most abundant protein in pancreatic juice during acute pancreatitis^23^. However, the release of REG3β in PDAC could be a less useful response since its interfering effect on EV uptake has different and even opposite responses depending on the target cell. We observed that cancer cell migration is increased in response to EVs generated by macrophages, thus the inhibitory effect of REG3β could be regarded as a protective response against tumor progression. By the contrary, EVs obtained from MIA PaCa-2 cells induced the switch of macrophages to an inflammatory M1 phenotype, an effect that was abolished by the interaction of EVs with REG3β. Avoiding or minimizing the M1 activation of macrophages may be a useful response in inflammatory processes as acute pancreatitis^24^, and could help to understand the high-level production of REG3β in this disease and the protective effect previously reported for this protein in different inflammatory situations^22, 25, 26^. Nevertheless, this binding effect seems to be probably counterproductive in tumoral processes since the inflammatory phenotype of macrophages promotes the immune reaction against the tumor and is considered a useful response^27^ opposed to the characteristic immunosupresor M2 phenotype of tumor-associated macrophages (TAMs)^28^. In this context, the presence of REG3β could be considered a pro-tumoral effect. The involvement of REG3β on macrophage polarization has been previously demonstrated^29^ although this is the first time that this effect is linked with the trafficking of EVs. Nevertheless, depending on tumor microenvironment composition, the amount of REG3β and the relevance of EV signaling, the effects of this lectin on tumor progression could be very different. This variability was evident when analyzing the impact of REG3β on the metabolic status of macrophages, tumor cells and CAFs.

In addition to interfering with EV signaling inside the tumor stroma, the interaction of these vesicles with REG3β also modifies their fate. In the absence of REG3β, EVs were easily incorporated to tumor cells of xenografts, while REG3β^+^ EVs remained at the intercellular space. As a consequence, it could be expected that these vesicles were finally removed from the tumor, thus reaching the circulatory system. In this line, we evaluated the presence of REG3β^+^ EVs in plasma of PDAC patients and, indeed, we confirmed that they have a greater amount of circulating EVs which are REG3β^+^. This is of importance since, in PDAC, it has been demonstrated a role for EVs on the generation of pre-metastatic niches in the liver^4^. Therefore, in the context of cancer, factors that promote the release of EVs into blood can be considered to play a prometastatic role and become a potential therapeutic target.

Our results also point the importance of the healthy tissue surrounding the tumor on establishing the specific characteristics of tumor microenvironment. Trafficking of EVs in PDAC could be affected by the presence of REG3β, but this effect can also be conditioned by changes in the production of this protein along the progression of the disease. Although high levels of REG3β are generated by pancreatic acinar cells, it is known that its synthesis is progressively inhibited during the development of pre-cancerous lesions along the PanIN stages, being completely absent in PDAC tumor cells^30^. Interestingly, at this point, the expression of REG3β is restricted to a limited layer of acinar cells around the tumor while neither the tumor cells nor the stroma generate this protein. This suggests that the distant microenvironment has the capability to interfere with cell-to-cell communication inside the tumor, thus modifying some of the features that characterize it. Altogether provide evidence of the importance of this mechanism in the cancer context, and emphasize the relevance of secreted lectins as a regulatory mechanism of internalization and functionality of EVs. Finally, it also seems reasonable to suggest that in diseases in which EVs have a significant role, REG3β or antibodies against REG3β could be used to modulate their effects.

In summary, our results indicate that, in PDAC, the physiological function of EVs could be markedly conditioned by the presence of REG3β in the tumor microenvironment. It is noteworthy how REG3β can interfere with the activity of EVs, modulating relevant cellular processes as migration, macrophage polarization or metabolic switch, the dysregulation of which is crucial in tumor progression. This binding mechanism highlights the need to take into account not only the content or functionality of EVs but also the context in which they are secreted. This seems to be a very important fact in PDAC, as the healthy tissue surrounding the tumor is committing the EV signaling and the pathophysiology of the disease.

## Material and Methods

### Cells

Human THP-1 cells were cultured in suspension in RPMI 1640 medium supplemented with 10% fetal bovine serum (FBS; GibcoTM, Thermo Fisher Scientific), 2 mM L-glutamine, 100 U/ml penicillin and 100 µg/ml streptomycin. Cells were differentiated to macrophages through a first incubation with 100 nM phorbol 12-myristate 13-acetate (PMA) (Sigma Aldrich) for 48 h. After that, the PMA-containing media was discarded and replaced with fresh media without PMA for a further 24 h.

Human pancreatic MIA PaCa-2 cells were cultured in DMEM medium supplemented with 10% FBS, 2 mM L-glutamine, 100 U/ml penicillin and 100 µg/ml streptomycin. The experiments were performed when 70% of confluence was achieved.

Cancer-associated fibroblasts (CAFs) were a primary culture isolated from pancreatic tissue of a PDAC patient. CAFs were cultured in serum-free defined medium supplemented with bovine pituitary extract (2%) and human epidermal growth factor (10 ng/ml).

All cells were grown in a humidified atmosphere of 95% air, 5% CO_2_ at 37 °C.

### EVs isolation

To isolate EVs from supernatants, it is necessary to use a culture media depleted from the EVs contained in FBS. To achieve this, RPMI or DMEM containing 20% FBS were ultracentrifuged at 120 000 × g for 16 h, filtered through a 0.22 µm syringe filter (Millipore) and 1:1 (v/v) mixed with non-supplemented RPMI or DMEM medium, respectively. Normal culture medium was replaced by EV-free medium 24 h before the experiments. In the case of CAFs, medium without BPE was used as EV-free medium.

For the EV isolation, supernatants were collected and centrifuged at 2 000 × g and 10 000 × g for 10 and 30 min, respectively, at 4 °C. The last supernatant was filtered through a 0.22 µm syringe filter in order to obtain the microvesicle fraction, and ultracentrifuged at 120 000 × g for 70 min. After that, the pelleted EVs were washed with phosphate-buffered saline (PBS) and centrifuged again at 120 000 × g. Quality of EV preparations was verified by electron microscopy analysis of their size and shape and by determining the presence/absence of CD81, TSG101, ALIX and Calnexin by Western Blot. The amount of EVs obtained was quantified by measuring their protein content using a Bradford assay, as previously described^31^.

### EVs staining

For internalization and binding assays, EVs were labeled with the PKH26 red fluorescent cell linker dye (Sigma Aldrich) for 5 min. The staining reaction was stopped with 3% BSA for 1 min. EVs were washed three times with PBS in order to remove the unbound dye, using 300 KDa Nanosep centrifugal devices (Pall Corporation).

### EVs uptake

To monitor EVs uptake, 3 µg/ml of labeled MPC-EVs were added to THP-1 macrophages in the presence of different concentrations of REG3β (0, 20, 100 and 500 ng/ml) for 45 min (Dynabio). The same experiment was performed with MIA PaCa-2 cells, which were incubated with labeled THP1-EVs for 2 h. REG3β concentrations used in this experiment were within the range of plasma REG3β levels reported in pancreatic cancer patients^32^. The concentration of EVs was selected according to previous *in vitro* studies^33, 34^.

EV internalization was analyzed by reading the amount of fluorescence with a fluorometric plate reader (Spectramax Gemini XS) and by fluorescence microscopy imaging.

### EV treatment with REG3β

Isolated EVs were divided into two groups. One of them was incubated with 500 ng/ml of REG3β (REG3β -treated EVs) for 30 min, while the corresponding volume of vehicle solution was added to the non-treated group. Both EV populations (REG3β-treated and non-treated) were incubated side by side under identical conditions to ensure that the only difference between them was the presence of REG3β. Then, the unbound protein was removed by three washing steps at 3500 × g with Nanosep 300 KDa filter devices. In all experiments, REG3β-treated EVs were compared with non-treated EVs.

### SDS-PAGE and Western Blot

EVs and cell proteins were extracted in RIPA buffer (10 mM Tris pH 8.0, 140 mM NaCl, 1% Triton X-100, 1 mM EDTA and 0.1% SDS) supplemented with protease inhibitors. The concentration of the isolated proteins was determined using a Bradford assay. 10 µg of protein were separated on a 12% SDS-PAGE and electrophoretically transferred under wet conditions onto a PVDF membrane (Immun-Blot, Bio Rad). Membranes were blocked for 1 h in 5% nonfat milk in PBS, followed by overnight incubation at 4 °C with antibodies against TSG101 (14497-1-AP; 1:1000), ALIX (12422-1-AP; 1:1000), Calnexin (10427-2-AP; 1:1000) (ProteinTech) and CD81 (10630D; 1:1000) (Invitrogen). Blots were washed and incubated for 1 h 30 min at room temperature with the DyLight 800-conjugated secondary antibody (1:10 000) (Thermo Scientific). Immunoreactive bands were visualized using an Odyssey Infrared Imaging System (LI-COR Biosciences).

### Binding assays

For the binding assays, 96-well black FLUOTRAC-600 plates (Greiner Bio-One) were coated with a specific anti-human REG3β antibody (Dynabio) overnight at 4 °C. The next day, plates were blocked with 3% BSA for 2 h at room temperature. Then, REG3β was diluted in the blocking solution and added for 1 h, followed by three washing steps. After that, stained EVs were added for 1 h in the presence of 1 mg/ml (5,5 mM) of mannose, 1 mg/ml of mannan or 5 mM of NAG (Sigma Aldrich), when needed. Finally, plates were washed to remove the unbound EVs and read on a Spectramax Gemini XS fluorimeter. All the steps were performed with mild agitation.

SureBeads™ Protein G Magnetic Beads (Bio-Rad) were used following the supplier’s specifications. Beads were coated with 50 µg/ml of anti-human REG3β antibody. Then, different concentrations of REG3β were added and the beads were placed in a rotator for 1 h. After washing, they were resuspended with stained EVs and further incubated for 1 h in the same conditions. The EVs were dissociated from the complex with an elution buffer (glycine 20 mM pH 2.0), and the amount of fluorescence of each condition was read on a fluorimeter.

### Macrophage polarization

Prior to the assay, MPC-EVs were incubated with 500 ng/ml of REG3β for 30 min, and the unbound protein was removed by three washing steps with Nanosep 300 KDa filter devices. The resulting blocked EVs (REG3β-blocked MPC-EVs) were used in the subsequent assay.

Macrophage-differentiated THP-1 cells were incubated with 500 ng/ml of REG3β, 3 µg/ml of non-treated MPC-EVs or 3 µg/ml of REG3β-blocked MPC-EVs. After 24 h, RNA was extracted using TRizol reagent (Life Technologies).

### RT-PCR and qPCR

Total RNA was extracted by phenol-chloroform extraction and ethanol precipitation using TRizol® reagent (Invitrogen). Isolated RNA was diluted in RNAse-free water and stored at −80 °C. RNA samples were quantified using a Nanodrop ND-1000 device. A reverse transcription reaction was performed on 1 μg RNA sample using iScript reagents (Bio Rad) and following the manufacturer’s specifications. The mixture was incubated at 25 °C for 5 min, 42 °C for 30 min, and 85 °C for 5 min. Finally, it was diluted in RNAse-free water so that the final concentration was 10 μg/ml and stored at −80 °C.

Subsequent qPCR amplification was performed using iTaq® SYBR Green Supermix (Bio Rad) and the corresponding primers listed in Table 1. The initial activation step of the Hot Start DNA polymerase (95 °C, 1 min 30 sec) was followed by 40 cycles of DNA amplification with fluorescence detection at the end of the elongation step as follows: denaturation at 95 °C (15 sec), annealing at 60 °C (30 sec), and synthesis at 72 °C (20 sec). Reactions were performed in duplicate and threshold cycle values were normalized to *GAPDH* gene expression. The specificity of the products was determined via a melting curve analysis. The ratio of the relative expression of target genes to *GAPDH* was calculated by using the ΔC(t) formula.

**Table 1:** Primers used for qPCR amplification.

Gene	Sequence
*GAPDH*	Forward: 5′-GATCATGAGCAATGCCTCCT-3′
	Reverse: 5′-TGTGGTCATGAGTCGTTCCA-3′
*IL1β*	Forward: 5′-GGACAAGCTGAGGAAGATGC-3′
	Reverse: 5′-TCGTTATCCCATGTGTCGAA-3′
*IL8*	Forward: 5′-ATTTCTGCAGCTCTGTGTGAAGGTGC-3′
	Reverse: 5′-TTGTGGATCCTGGCTAGCAGAC-3′
*CXCL2*	Forward: 5′-AATCACCAGCAGCAAGTGTC-3′
	Reverse: 5′-TGGGTTGTGGAGTGAGTGTT-3′
*CCL2*	Forward: 5′-CAAACTGAAGCTCGCACTCTCGCC-3′
	Reverse: 5′-ATTCTTGGGTTGTGGAGTGAGTGTTCA-3′
*MRC1*	Forward: 5′-GGATGGATGGCTCTGGTG-3′
	Reverse: 5′-TCTGGTAGGAAACGCTGGT-3′
*TGFβ*	Forward: 5′-GTGGAAACCCACAACGAAAT-3′
	Reverse: 5′-CACGTGCTGTCTCACTTTTA-3′

### Migration assay

Prior to the assay, THP1-EVs were incubated with 500 ng/ml of REG3β for 30 min, and the unbound protein was removed by three washing steps with Nanosep 300 KDa filter devices. The resulting blocked EVs (REG3β-blocked THP1-EVs) were used in the subsequent assay.

MIA PaCa-2 cells were grown until confluence and treated with 0,5 µg/ml of mitomycin C (Roche) 2 h before the experiment to arrest proliferation. Then, the scratch was made with a pipette tip and cells were incubated with 500 ng/ml of REG3β, 3 µg/ml of non-treated THP1-EVs or 3 µg/ml of REG3β-blocked THP1-EVs. After 24 h, wound area was measured and cell migration quantified using Cell^R software.

### Metabolomic analysis

Metabolomic analyses were performed by OWL Metabolomics (Bizkaia, Spain). Four ultra-high performance liquid chromatography coupled to time-of-flight mass spectrometry (UHPLC-ToF-MS)-based platforms were used for optimal profiling of the cellular metabolome. Cell pellets from three experiments were pooled, resuspended in cold water and vortexed. Proteins were precipitated from the lysed cell samples by adding 800 µl methanol. After short vortex mixing, the samples were spiked with chloroform. Both extraction solvents were spiked with metabolites not detected in unspiked cell extracts. Then, samples were incubated at −20 °C for 30 minutes and after vortexing them, 500 µl were collected to be analyzed in each UHPLC-MS platform^35^. Platform 1 for Fatty acyls, bile acids, steroids and lysoglycerophospholipids. Platform 2 for Glycerolipids, cholesteryl esters, sphingolipids and glycerophospholipids. Platform 3 for Amino acids profiling and Platform 4 for Polar metabolites profiling, including Central carbon metabolism.

All data were processed using the TargetLynx application manager for MassLynx 4.1 software (Waters Corp., Milford, USA). A set of predefined retention time, mass-to-charge ratio pairs, Rt-m/z, corresponding to metabolites included in the analysis are fed into the program. Associated extracted ion chromatograms (mass tolerance window = 0.05 Da) are then peak-detected and noise-reduced in both the LC and MS domains such that only true metabolite related features are processed by the software. A list of chromatographic peak areas is then generated for each sample injection. The peak detection process included 408 LC–MS features, identified prior to the analysis. A moving average smoothing method was applied for noise reduction. Normalization factors were calculated for each metabolite by dividing their intensities in each sample by the recorded intensity of an appropriate internal standard in that same sample^36^. Further normalization procedure was applied by dividing every sample by its protein content, as part of the biological normalization. All calculations were performed using statistical software package R v.3.1.1 (R Development Core Team, 2011; http://cran.r-project.org).

### Electron microscopy and immunogold labeling

Isolated EVs were fixed in 2% paraformaldehyde, adsorbed in formvar-coated nickel grids for 20 min and negative stained with 4% uranyl oxalate. Grids were air dried and observed in a JEOL-1010 Transmission Electron Microscope at 80 kV.

For the immunogold labeling, grids were blocked with 1% BSA for 10 min, followed by anti-human REG3β antibody incubation for 1 h and 12 nm gold-conjugated goat anti-rabbit IgG secondary antibody incubation (Jackson Laboratories) for 30 min. Then, samples were further fixed with 1% glutaraldehyde, counterstained with 4% uranyl oxalate and embedded with a mixture of 2% methyl cellulose - 4% uranyl acetate.

### Subcutaneous tumor xenografts

Tumors were induced by subcutaneous injection of 20 × 10^6^ MIA PaCa-2 cells in PBS supplemented with 0.1% glucose in 4 six-week-old male BALB/C swiss nude immunodeficient mice. Four weeks after cell injection, 5 µg of PKH26-labeled EVs purified from cultured MIA PaCa-2 cells were treated or non-treated with REG3β and injected into the tumors. One hour later, mice were sacrificed, tumors were fixed and analyzed for the location of EVs by fluorescent microscopy.

### Plasma from PDAC patients and healthy donors

Plasma samples obtained from patients diagnosed of PDAC (n = 15) or healthy volunteers without personal history of any cancer (n = 15) were used to separate EVs. Patients were recruited from Hospital Clinic of Barcelona (Catalonia, Spain) and blood samples were obtained before any treatment was applied to the patients. The study was approved by the Institutional Ethics Committee of this Institution, and written informed consent was obtained from all participants in accordance with the Declaration of Helsinki.

10 ml of whole blood from each participant were collected in EDTA tubes. Blood samples were placed at 4 °C until plasma separation, and plasma was frozen within 6 h of the blood draw. Briefly, samples were centrifuged at 1,600 × g for 10 min at 4 °C to spin down blood cells, and plasma was transferred into new tubes, followed by further centrifugation at 16,000 × g for 10 minutes at 4 °C to completely remove cellular components. Plasma was then aliquoted and stored at −80 °C until use.

### Immunolocalization of REG3β in human PDAC

Pancreatic sections were fixed in 4% paraformaldehyde and embedded in paraffin. Sections were probed with the primary antibody against REG3β and revealed by goat anti-rabbit IgG secondary antibody horseradish peroxidase (HRP)-conjugate. Samples were examined with a Nikon Eclipse 90i microscope.

### Statistics

Statistical analysis was performed with Graphpad Prism software. Data are presented as mean ± SEM. Differences between groups were analysed using a two-tailed Student’s t-test for comparison of two groups, and by One-way analysis of variance (ANOVA) followed by Tukey’s post-test when comparing three or more groups. Statistical significance was considered when p < 0.05. For binding experiments, EVs binding rates were fit to one-site binding hyperbola models. R^2^ quantifies goodness-of-fit. For experiments performed with human plasma samples, the two-tailed Mann–Whitney U test was used.
